# Exploring clinically relevant risk profiles in patients undergoing lumbar spinal fusion: a cohort study

**DOI:** 10.1007/s00586-022-07325-5

**Published:** 2022-07-28

**Authors:** Esther R. C. Janssen, F. G. Prestigiacomo, E. A. P. van Leent, N. L. U. van Meeteren, M. Hulsbosch

**Affiliations:** 1 Department of Orthopedic Surgery, VieCuri Medical Centre, Tegelseweg 210, 5912BL Venlo, The Netherlands; 2 Department of Orthopedics and Research School Caphri, Maastricht University Medical Centre+, Maastricht, The Netherlands; 3 Top Sector Life Sciences and Health (Health~Holland), The Hague, The Netherlands; 4 Department of Anesthesiology, Erasmus Medical Centre, Rotterdam, The Netherlands; 5 Topcare, Leiden, The Netherlands

**Keywords:** Latent class analysis, Fitness, Spine surgery, Lumbar spine, Prognosis

## Abstract

**Purpose:**

To explore risk profiles of patients scheduled for lumbar spinal fusion (LSF) and their association with short-term recovery of patient after surgery.

**Methods:**

Forty-nine patients scheduled for elective 1–3 level LSF between March 2019 and June 2020 were included. Patients underwent a preoperative risk screening, consisting of an anamnesis, questionnaires and physical performance tests. A latent profile analysis (LPA) was used to identify possible risk profiles within this population.

**Results:**

Two risk profiles could be established: a fit and deconditioned risk profile. A significant between-profile difference was found in smoking status (*p* = 0.007), RAND36-PCS (*p* < 0.001), Timed Up and Go (TUG) (*p* < 0.001), de Morton Morbidity Index (DEMMI) (*p* < 0.001), finger floor distance (*p* = 0.050), motor control (*p* = 0.020) and steep ramp test (*p* = 0.005). Moreover, the fit risk profile had a significant shorter time to functional recovery (3.65 days versus 4.89 days, *p* = 0.013) and length of hospital stay (5.06 days versus 6.00 days, *p* = 0.008) compared to the deconditioned risk profile. No differences in complication rates between both risk profiles could be established. Allocation to a risk profile was associated with the functional recovery rate (*p* = 0.042), but not with LOS or complications.

**Conclusion:**

This study found a fit and deconditioned risk profile. The patients with a fit risk profile perceived a better quality of life, performed better in mobility, motor control, cardiopulmonary tests and showed also a significant shorter stay in the hospital and a shorter time to functional recovery. Preoperatively establishing a patient’s risk profile could aid in perioperative care planning and preoperative decision-making.

**Supplementary Information:**

The online version contains supplementary material available at 10.1007/s00586-022-07325-5.

## Introduction

Chronic low back pain (LBP) is often associated with degenerative disorders of the lumbar spine [1, 2]. If conservative treatment fails for people with degenerative disorders of the lumbar spine, lumbar spinal fusion (LSF) could be considered, particularly when patients experience neurogenic claudication. Undergoing any surgical procedure, like LSF, is a major life event and includes risks for adverse events such as prolonged recovery and complications [3]. Patients suffering from chronic LBP considering LSF often already have had a period of decreased physical activity (deconditioning). If this vicious cycle continuous in the long term this often contributes to chronicity of pain and further deconditioning [4]. A decreased level in physical activity negatively affects cardiovascular capacity, muscle strength and motor control [4]. Moreover, passive coping strategies, avoidant behaviour, catastrophizing and hypervigilance negatively influence pain level, mobility and muscle awareness, further contributing to deconditioning [5]. Patients opting for LSF are probably at various stages of deconditioning and those who are more deconditioned may be at increased risk of negative postoperative outcomes [6–8].

In clinical practice, patients eligible for LSF are often divided into subgroups by their medical diagnosis, in order to estimate the success probability after surgery [9]. Previous research showed various risk factors are also important to consider when estimating a person’s postoperative outcomes after LSF: male gender, young age, working people, non-smokers and high income are predictors of a good outcome [10, 11]. However, relying on these variables alone to categorize patients into risk profiles foregoes other important information related to postoperative success chance, as the population undergoing LSF is rather heterogeneous.

Measuring the stage of deconditioning of patients eligible for LSF may provide us with important additional information to estimate postoperative success probability, but its importance is still unclear. Using physical fitness to measure deconditioning in addition to the aforementioned risk factors may enable us to identify clinically relevant risk profiles in patients eligible for LSF. Patients falling within these risk profiles may have unique clinical needs, and the risk profiles could be related to short- and long-term postoperative outcomes [12]. Therefore, preoperatively identifying a patient’s risk profile can help surgeons and patients with preoperatively estimating a person’s success probability, expectation management and perioperative care planning. Consequently, we may in the near future be able to allocate care resources more efficiently.

Therefore, the aim of this study is to explore clinically relevant risk profiles within the population of patients undergoing a LSF and establish the association of these risk profiles with short-term outcomes after LSF.

## Methods

### Study setting

This prospective single-centre cohort study was performed between March 2019 and June 2020. Inclusion criteria were: (1) patients > 17 years with degenerative disc disease of the lumbar spine, (2) patients scheduled for elective 1–3 level LSF and (3) good understanding of the Dutch language, written and verbal. Exclusion criteria were: (1) missing preoperative physical screening and (2) missing follow-up data. Degenerative disc disease was defined as: spondylolisthesis, degenerative disc disease without listhesis or prior spine surgery (laminectomy or LSF). Methods of LSF performed in this hospital were the posterior lumbar interbody fusion (PLIF) and transforaminal lumbar interbody fusion (TLIF). A non-WMO statement, waiver of approval statement, was obtained from the local Medical Ethical Committee (2019–1052).

### Preoperative screening

Preoperative risk screening was part of standard care and consisted of an anamnesis, questionnaires and a physical performance test battery.

Questionnaires on patient’s perceptions of physical functioning, using the Oswestry Disability Index (ODI), pain, using the visual analogue scale (VAS) and health-related quality of life, using the RAND-36 (physical component score (PCS) and mental component score (MCS)) were administered.

Physical performance tests executed by patients were supervised by physiotherapists. Five constructs were measured; (1) aerobic capacity, (2) muscle strength of the lumbar spine, (3) motor control of the lumbar spine, (4) flexibility and (5) mobility. For testing the aerobic capacity, the steep ramp test was used. The Sorensen test used to measure the muscle strength of the lumbar spine. Motor control was measured with four tests: the sitting knee extension test, the posterior pelvic tilt test, the waiter’s bow test and the one-leg stance test. To objectify mobility we used the timed “up and go” test (TUG) and de Morton Mobility Index (DEMMI). The finger-floor distance test was used to reflect flexibility of the lumbar spine. The screening protocol can be found in (see online Appendix 1).

### Outcome variables

Short-term outcomes after LSF were used to analyse whether the discovered risk profiles were related to postoperative clinical course. Postoperative inpatient functional recovery was measured using the modified Iowa Level of Assistance Scale (mILAS) every day during admission, which is a measurement tool to assess capability to safely perform five activities of daily life [13]. A mILAS score of 0 showed the patient was functionally recovered. The time in days till achieving a mILAS of 0 was used to quantify inpatient functional recovery. Moreover, length of stay (LOS) and complications were considered postoperatively. Postoperative complications were scored as major if the complication was potentially life threatening, needed a second surgery or caused considerable suffering which lead to prolonged recovery. Complications were scored as minor if they were reversible minor events which had no consequence for the recovery process [14].

### Statistical analysis

Statistical analysis was performed using SPSS software version 26 (IBM Corp., IBM SPSS Statistics for Windows, Armonk, NY, USA) for data cleaning and descriptive analyses and R (R Foundation for Statistical Computing, Vienna, Austria) for performing the latent profile analysis (LPA).

Baseline variables of participants allocated to each profile were compared using an independent sample *t*-test or its non-parametric equivalent (Mann–Whitney *U*-test) with Bonferroni corrections. The same analyses were used to compare postoperative outcomes between identified profiles. Single stochastic regression imputation was used to impute any missing data in baseline variables. We used LPA to explore if risk profiles could be established, based on the input variables. With executing a LPA, it is possible to develop meaningful typologies or risk profiles that are similar in their responses to measured variables within the population. Lower values of the Bayesian information criterion, Akaike information criterion and log likelihood indicate a better model fit in LPA. Using these statistics, the optimal number of risk profiles was determined, starting with one profile than two, and so on. Descriptive names for different risk profiles were selected, based on clinical expertise to reflect their clinical relevance. Univariate linear regression modelling was used to determine whether risk profile allocation was associated with postoperative outcomes. Statistical tests were considered significant if *p* < 0.05.

## Results

In total 72 patients underwent LSF between March 2019 and June 2020, 23 patients were excluded due to missing screening data, being deceased, undergoing non-elective surgery or missing outcome data. Forty–nine participants reached the inclusion criteria and were eligible for analysis. The baseline characteristics of the population are shown in Table 1. Baseline characteristics did not have strong correlation *r* < 0.5.

**Table 1:** Baseline characteristics, patient-reported outcome measurements (PROMs), physical screening and outcomes

Variables	Patient group			
	*N*	Mean	SD	%
Patient characteristics				
Age	49	61.31	11.94	
Sex (%female)	49			71
BMI	49	28.34	6.02	
ASA (% I and II)	49			63
Smoking (yes)	49			29
Surgery characteristics				
Surgery indication: Deg. Listhesis Deg. without listhesis Prior spine surgery	49			57 25 18
Number of levels fused 1 level 2 levels 3 levels	49			65 27 8
Level fused L5–S1 L4–L5 L4–S1 L3–L5 L3–S1 L2–L3 L2–L4 L2–L5 L1–L3 L1–L4				24 31 18 4 4 8 4 2 2 2
Surgery type: PLIF	49			20
TLIF				80
Postoperative complications (≤ 30 days)				
Minor complications Urinary tract infection Postoperative atrial fibrillation Heavy wound leakage	49			2 2 2
Major complications Malposition screw Wound leakage and additional decompression Lumbar infection debridement	49			2 2 8
Patient-reported outcome measurements (PROMs)				
Maximum pain (range 0–100)	43	78.07	15.13	
RAND-36 physical component sum score (range 0–100)	44	26.32	6.16	
RAND-36 mental component sum score (range 0–100)	44	42.21	12.60	
Oswestry Disability Index (range 0–100)	44	54.50	11.01	
Physical screening				
Steep Ramp peak (Watt/kg)	42	2.32	0.90	
Motor control test (n correct test out of 4)	49	1.88	1.24	
Mobility (TUG, in sec.)	47	9.15	4.19	
Flexibility (finger floor distance, in cm)	49	13.21	13.89	
Endurance of strength of lumbar spine muscle (time in sec.)	12	22.10	16.60	
Outcomes				
Functional recovery (mILAS 0 in days)	49	4.10	2.07	
Length of stay (in days, incl. surgery day)	49	5.41	2.78	
Complications (% complications)	49			18

ASA; American Society of Anesthesiologists, BMI; body mass index, cm; centimetres, deg.;degenerative, incl.; including, mILAS; modified Iowa Level of Assistance Scale, PLIF; posterior lumbar interbody fusion, RAND; Research and Development, sec.; seconds, SD; standard deviation, TLIF; transforaminal lumbar interbody fusion, TUG; Time Up and Go

In total 9 complications were registered after LSF and 40 patients (81.6%) had no complications after surgery. The three (6.1%) minor complications comprised: urinary tract infection (*n* = 1), postoperative atrial fibrillation (*n* = 1) and wound leakage (*n* = 1). Of the six (12.2%) patients with a major complications, four showed a lumbar infection and needed a debridement, one had screw malposition, and one had wound leakage requiring additional treatment.

### Latent profile analysis

Performing the LPA, the model with two profiles showed a decreased AIC, BIC and log likelihood which indicate a better performance than the single profile model (Table 2). The three profiles model may perform even better when looking at the AIC and log likelihood, but not when considering the BIC. Therefore, the two profiles model is preferred over the three profiles model, both models were analysed in their association with the postoperative clinical course. A four profiles model was not considered as the performance deteriorated on all performance measures. Moreover, within profile cluster sizes were considered too small < 10.

**Table 2:** Performance of latent profile analysis

*N* classes	AIC	BIC	Log likelihood	% of total population in each class		
				Profile 1	Profile 2	Profile 3
1	3748	3805	− 1844	100%		
2	3684	3799	− 1781	65%	35%	
3	3676	3850	− 1746	55%	25%	20%

*AIC*; Akaike information criterion, *BIC*; Bayesian information criterion

### Description of profiles

Using the two profile model of the LPA, a significant between-profile difference in smoking status (*p* = 0.007), RAND36-PCS (*p* < 0.001), TUG (*p* < 0.001), DEMMI (*p* < 0.001), finger floor distance (*p* = 0.05), motor control (*p* = 0.02) and steep ramp test (*p* = 0.005) was found (Table 3). Due to these differences, the risk profiles were defined as a fit risk profile (profile 1) and a deconditioned risk profile (profile 2).The fit risk profile had a shorter time to functional recovery and length of hospital stay than the deconditioned risk profile. There was no significant differences in the complication rate between the two profiles. Major complications occurred 3 times (16.7%) in the first class and 4 times (12.9%) in the second profile. Allocation to a risk profile was a predictor for the functional recovery rate (*p* = 0.042), but not for LOS or complications (Table 4).

**Table 3:** Two profile model of the LPA. Baseline characteristics of populations in latent profiles and between profile differences

Variable	Profile 1 (fit profile) (*n* = 31), mean (SD)/N (%)	Profile 2 (deconditioned profile) (*n* = 18), mean (SD)/N (%)	Between profile difference*
Age	60.52 (12.70)	62.67 (10.71)	0.803ˣ
Diagnostic category Deg. with listhesis Deg. without listhesis Prior spine surgery	20 (64.5) 7 (22.6) 4 (12.9)	8 (44.4) 5 (27.8) 5 (27.8)	0.139ˣ
Sex Female Male	23 (74.2) 8 (25.8)	12 (66.7) 6 (33.3)	0.578ˣ
ASA I–II III	19 (61.3) 12 (38.7)	12 (66.7) 6 (33.3)	0.710ˣ
BMI	27.29 (5.16)	30.15 (7.06)	0.085ˣ
Smoking Yes No	13 (41.9) 18 (58.1)	1 (5.6) 17 (94.4)	0.007*ˣ
VAS maximal pain	73.34 (16.80)	79.89 (13.74)	0.167ˠ
ODI	52.81 (13.92)	57.89 (6.84)	0.155ˠ
RAND-36 PCS	29.10 (5.70)	22.35 (4.15)	< 0.001*ˠ
RAND-36 MCS	42.21 (11.80)	41.99 (12.51)	0.950ˠ
TUG (sec.)	7.08 (1.30)	12.81 (5.02)	< 0.001*ˠ
DEMMI	17.55 (1.06)	15.33 (2.22)	< 0.001*ˣ
Finger Floor distance (cm)	10.69 (13.71)	17.56 (13.46)	0.050*ˣ
Motor control (correct out of 4) 0 1 2 3 4	3 (9.7) 8 (25.8) 6 (19.4) 8 (25.8) 6 (19.4)	2 (11.1) 10 (55.6) 5 (27.8) 1 (5.6)	0.020*ˣ
Sorensen performed Yes No	12 (38.7) 19 (61.3)	0 (0) 18 (100)	0.003*ˣ
Steep ramp test (Watt/kg)	2.80 (1.08)	1.83 (0.98)	0.005*ˣ
Time to functional recovery (days)	3.65 (1.84)	4.89 (2.27)	0.013*ˣ
LOS (days)	5.06 (3.23)	6.00 (1.68)	0.008*ˣ
Complications None Minor Major	14 (77.8) 1 (5.6) 3 (16.7)	25 (80.6) 2 (6.5) 4 (12.9)	0.790ˣ

ASA; American Society of Anesthesiologists, BMI; body mass index, cm; centimetres, deg.;degenerative, incl.; including, DEMMI; de Motor Mobility Index, mILAS; modified Iowa Level of Assistance Scale, ODI; Oswestry Disability Index, RAND-PCS/MCS; Research and Development Physical/Mental Component Score, sec.; seconds, TUG; Time Up and Go, VAS; visual Analogue scale

*Significant

ˣMann–Whitney *U*-test

ˠIndependent sample *t*-test

**Table 4:** Univariate predictive performance of profile differentiation in two profiles in functional recovery, length of hospital stay, complications using linear and logistic regression

	Functional recovery (lin.)	LOS (lin.)	Complications (log.)
R2	0.085	0.027	0.002
*B*	1.244	0.935	0.174
*P* value	0.042*	0.261	0.810

*Significant

The three profiles LPA showed significant differences between the three profiles on: smoking (*p* = 0.009), ODI (*p* < 0.001), RAND-36 MCS (*p* = 0.010), RAND-36 PCS (*p* = 0.002), TUG (*p* < 0.001), DEMMI (*p* = 0.004), finger floor distance (*p* = 0.009) and LOS (*p* = 0.019) (see Online Appendix 2). A non-significant difference was found on pain (*p* = 0.06) between profiles with a clinically relevant difference of 12.48 points on the VAS between profile one and three. Due to these differences, the risk profiles were defined as fit risk profile (profile 1), deconditioned risk profile (profile 2) and intermediate risk profile (profile 3). Allocation to a risk profile in the three profile model was not predictive for postoperative outcomes (see online Appendix 3).

## Discussion

This study aimed to explore possible risk profiles within the population of patients undergoing a LSF and if these risk profiles were associated with short-term postoperative outcomes. This study was performed with an explorative intention to investigate whether measuring deconditioning preoperatively is worthwhile and if risk profiles should be investigated further. Two risk profiles could be established; a fit and deconditioned risk profile, showing differences in smoking status, RAND36-PCS, TUG, DEMMI, finger floor distance, motor control and the steep ramp test. The fit risk profile had a shorter LOS and a shorter time to functional recovery. Risk profile allocation was associated with time to functional recovery, but not with LOS or complications.

To the best of our knowledge, no preoperative risk profiles in this population have been established. Establishing such risk profiles is valuable as heterogeneity in the patients eligible for LSF is well recognised [15]. In patients with low back pain, risk profile approaches have already been proposed and give patients together with their health professionals the opportunity to make treatment choices according to these profiles. In other surgical populations, risk profiles are also defined to help in the decision-making process, for example, in patients undergoing total knee arthroplasty, a persistent pain class and poor function class could be identified, similar to our deconditioned risk profile [16].

Evidence shows the importance of deconditioning for establishing risk profiles and estimating surgical outcomes for major surgery. Snowden et al. [17] showed that patients with a higher anaerobic threshold were defined as low-risk population and showed successful surgical outcomes and lowered hospital stay and costs in hepatobiliary surgery. Physical fitness is an important predictive variable in major surgery like cardiac, oncological and abdominal surgery, which likely also holds true for LSF [18–20]. This seems logical, as good physical fitness or cardiorespiratory capacity is an important aspect of high physiological reserve necessary to adequately deal with surgery-induced stress [21]. Moreover, this hypothesis strongly corresponds with the views of our orthopaedic surgeons, as they also recognize that fitter patients generally recover faster after surgery.

Surprisingly, the fit risk profile comprised more active smokers than the deconditioned risk profile. However, we consider it unlikely that fit patients smoke more frequently, as it would contradict evidence on both lifestyle epidemiology and associations of smoking with outcomes after (spinal) surgery [10, 11, 22, 23]. Moreover, no difference in complication rates could be found between the risk profiles. Due to the relatively small sample size, the low number of smokers and complications within 30 days after LSF, these findings may be coincidental.

Only 25% of the patients performed the Sorensen test. The reason for missingness in the Sorensen test was mainly unwillingness or being unable to perform the test. Patients who did not perform the Sorensen test, mostly originated from the deconditioned group, according to their other characteristics. Due to their deconditioning, they may not have had enough muscle strength to perform this test. Physiotherapists supervising the screening confirmed that patients could mostly not perform the test due to lack of strength or for fear of pain when performing the test. Therefore, a more accessible alternative to measuring muscle strength in these patients is advocated. Based on the literature, we would recommend hand grip strength or sarcopenia measures, as these were related to postoperative outcomes in other types of major surgery [24, 25].

### Strengths and limitations

Several strengths and limitations were apparent in our study. A strength of our study is the use of a wide variety of preoperative measurements to identify relevant risk profiles, with the aid of clinical judgement to interpret the risk profiles. By doing so, we were able to identify clinically relevant risk profiles based on characteristics, such as physical fitness, previously often left out of scope. The sample represents a true population of patients eligible for LSF seen in clinical practice, due to the minimal exclusion criteria. This is especially important when we want to identify clinically relevant risk profiles in a heterogeneous population. Limitation of this study is the sample size. Due to COVID-19, our inclusion was limited. Unfortunately during this period, preoperative risk screening had to be cancelled, which led to exclusion of these patients from our analysis. The literature is not uniform in its recommendation on sample size calculation for LCA and is dependent on the complexity and correlation between classes. Nylund–Gibson suggests that *n* = 300 is desirable, however, for less complex models, a smaller sample size may be sufficient. For a follow-up study, we would suggest a sample approximating *n* = 300 [26]. Due to the explorative nature and the relatively small sample of this study, our study may not be generalizable, but should be viewed as an explorative study guiding a research topic worthy of validation in future studies.

### Clinical implications

Until now perioperative care for patients is often still one-size-fits all. Implementing risk profiles into clinical practice can help frame a more preventive patient-specific approach to perioperative care for patients opting for LSF. If these risk profiles are validated, the next step could be identifying specific perioperative treatment plans matched to these risk profiles to better manage hospital resources and of course fit patient’s needs. The fit cluster could be scheduled for short stay surgery procedures, whilst the deconditioned risk profiles may benefit from prehabilitation strategies, such as exercise therapy, reducing their risk of prolonged functional recovery and hospital length of stay. In turn, we should explore if these optimized perioperative pathways could improve postoperative outcomes.

## Conclusion

In the population eligible for LSF, a fit and deconditioned risk profiles could be identified. A preoperative risk screening including a physical fitness screening for patients eligible for LSF can be useful to identify these clinically relevant subgroups. We should validate these risk profiles and probably tailor care to patients falling within these different risk profiles, as they may have different healthcare needs in the run-up time to surgery.

## Supplementary Information

Below is the link to the electronic supplementary material.

Supplementary file1 (DOCX 15 KB)

Supplementary file2 (DOCX 12 KB)

Supplementary file3 (DOCX 1613 KB)

## Data Availability

The datasets generated during and/or analysed during the current study are available from the corresponding author on reasonable request.

## Code availability

Statistics code is available upon request.
