# Supplementary material for: Exploring clinically relevant risk profiles in patients undergoing lumbar spinal fusion: a cohort study
Source: Eur Spine J. 2022 Jul 28;31(10):2473–80. doi: 10.1007/s00586-022-07325-5 (PMC9333351; doi:10.1007/s00586-022-07325-5)
Supplement: Supplementary file 1 — Supplementary file1 (DOCX 15 KB) [file 586_2022_7325_MOESM1_ESM.docx]

***Appendix 1.*** Latent classes analysis with 3 classes

**Table 5.** Baseline characteristics of populations in latent classes and between 3 classes differences

| **Variable** | **Profile 1 (fit profile) (n=27), mean (SD)/ N (%)** | **Profile 2 (deconditioned profile) (n=12), mean (SD)/ N (%)** | **Profile 3 (intermediate profile)(n=10), mean (SD)/ N (%)** | **Between Profile difference*** |
| --- | --- | --- | --- | --- |
| Age | 64.67 (7.92) | 61.08 (10.66) | 52.50 (17.88) | 0.109 |
| Diagnostic category  deg. with listhesis  deg. without listhesis  prior spine surgery | 16 (59.3)  8 (29.6)  3 (11.1) | 3 (25.0)  4 (33.3)  5 (41.7) | 9 (90.0)  0  1 (10.0) |  |
| Sex  Female  Male | 20 (74.1)  7 (25.9) | 10 (83.3)  2 (16.7) | 5 (50.0)  5 (50.0) | 0.211 |
| ASA  I-II  III | 17 (63.0)  10 (37.0) | 7 (58.3)  5 (41.7) | 7 (70.0)  3 (30.0) | 0.854 |
| BMI | 27.36 (5.38) | 31.35 (7.43) | 27.39 (5.13) | 0.148 |
| Smoking  Yes  No | 8 (29.6)  19 (70.4) | 0  12 (100.0) | 6 (60.0)  4 (40.0) | 0.009 |
| VAS maximal pain | 71.59 (15.77) | 78.17 (16.13) | 84.07 (13.36) | 0.060 |
| ODI | 49.35 (10.16) | 54.83 (4.55) | 68.85 (11.36) | <0.001 |
| RAND-36 PCS | 28.24 (6.39) | 22.18 (3.80) | 27.58 (5.28) | 0.010 |
| RAND-36 MCS | 45.95 (11.59) | 42.81 (10.71) | 30.98 (7.02) | 0.002 |
| TUG (sec.) | 7.08 (1.40) | 12.93 (8.14) | 10.36 (4.84) | <0.001 |
| DEMMI | 17.52 (0.98) | 14.92 (2.40) | 16.8 (1.8) | 0.004 |
| Finger Floor distance (cm) | 7.24 (7.49) | 16.00 (10.30) | 26.00 (20.92) | 0.009 |
| Motor Control (correct out of 4)  0  1  2  3  4 | 3 (11.1)  8 (29.6)  4 (14.8)  6 (22.2)  6 (22.2) | 0  7 (58.3)  4 (33.3)  0  1 (8.3) | 2 (20.0)  3 (30.0)  3 (30.0)  2 (20.0)  0 | 0.313 |
| Steep ramp test (Watt/kg) | 2.66 (0.89) | 1.86 (1.06) | 2.55 (1.63) | 0.124 |
| Time to functional recovery (days) | 3.85 (2.03) | 4.83 (2.52) | 3.90 (1.52) | 0.230 |
| LOS (days) | 4.85 (2.05) | 6.33 (1.37) | 5.80 (4.96) | 0.019 |
| Complications  None  Minor  Major | 22 (81.5)  3 (11.1)  2 (7.4) | 9 (75.0)  0  3 (25.0) | 8 (80.0)  0  2 (20.0) | 0.811 |

*Kruskal Wallis test

**Abbrevations:**  ASA; American Society of Anesthesiologists, BMI; Body Mass Index, cm; centimetres, deg.;degenerative, incl.; including, DEMMI; de Motor Mobility Index, mILAS; modified Iowa Level of Asisstance Scale, ODI; Owestry Disability Index, RAND-PCS/MCS; Research and Development Physical/Mental Component Score, sec.; seconds , TUG; Time Up and Go, VAS; Visual Analog Scale
