# Supplementary material for: Exploring clinically relevant risk profiles in patients undergoing lumbar spinal fusion: a cohort study
Source: Eur Spine J. 2022 Jul 28;31(10):2473–80. doi: 10.1007/s00586-022-07325-5 (PMC9333351; doi:10.1007/s00586-022-07325-5)
Supplement: Supplementary file 2 — Supplementary file2 (DOCX 12 KB) [file 586_2022_7325_MOESM2_ESM.docx]

***Appendix 2.*** Univariate predictive performance of profile differentiation

**Table 6.** Univariate predictive performance of profile differentiation in three classes in functional recovery, length of hospital stay, complications using linear and logistic regression.

|  | **Functional recovery (lin.)** | **LOS (lin.)** | **Complications (log.)** |
| --- | --- | --- | --- |
| R2 | 0.003 | 0.031 | 0.007 |
| B | 0.152 | 0.609 | -0.095/0.288 |
| P-value | 0.687 | 0.226 | 0.919-0.781 |

*significant
