# Supplementary material for: Exploring clinically relevant risk profiles in patients undergoing lumbar spinal fusion: a cohort study
Source: Eur Spine J. 2022 Jul 28;31(10):2473–80. doi: 10.1007/s00586-022-07325-5 (PMC9333351; doi:10.1007/s00586-022-07325-5)
Supplement: Supplementary file 3 — Supplementary file3 (DOCX 1613 KB) [file 586_2022_7325_MOESM3_ESM.docx]

**Appendix 3. Screening protocol**

Physical performance measures: Pre-operative I) movement control, II) back muscle strength, III) aerobic capacity, IV) flexibility and V) functional capacity are assessed by a trained physical therapist. Physical performance screening takes place a couple of weeks before undergoing lumbar spinal fusion.

Ad I) *Movement control*: Sitting one leg knee extension, posterior pelvic tilt, waiter's bow and the one leg stance test are considered reliable tests for examining movement control of the lumbar spine.^1^ Although multiple tests were presented in the protocol of Luomajoke et al., these test showed the highest reliability values (kappa >0.6).^1^ Moreover, all tests are easy to perform and are therefore appropriate for use in daily practice. The tests will be rated by a physical therapists as “*correct*” or “*not correct*”.

- One leg knee extension: in sitting position the patient is asked to extend one leg at least 30^o^ while holding the spine in a stable position. The test is graded as "correct" when the patient is able to extend the knee without flexion in the lumbar spine.


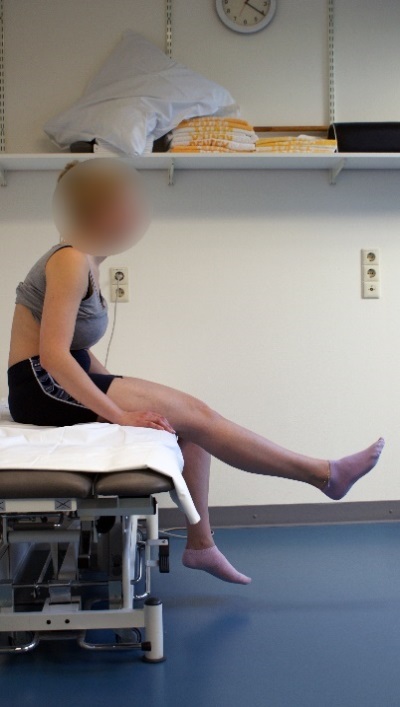


Incorrect


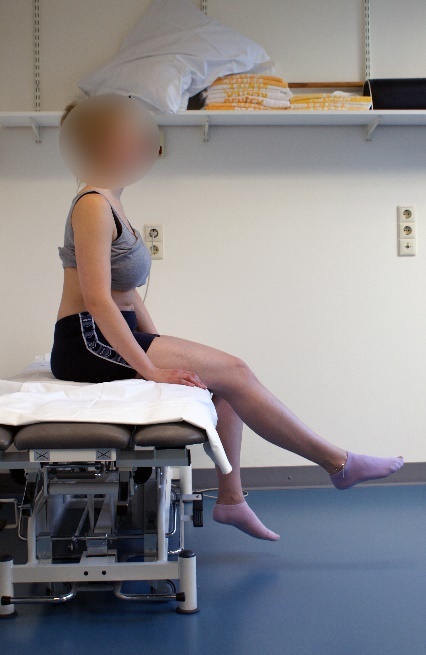


Correct

- Posterior pelvic tilt: while standing, the patient is asked to tilt the pelvis backward. The test is graded as "correct" when the patient is able to tilt the pelvis dorsally.


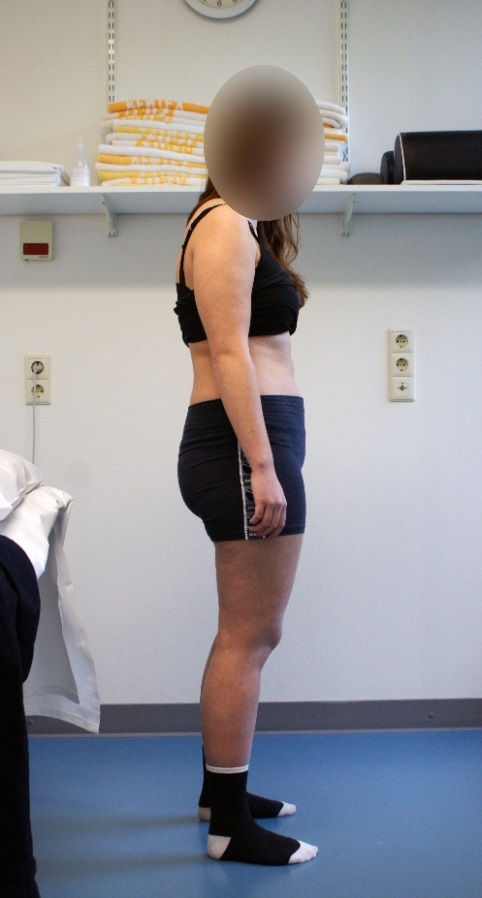


Correct


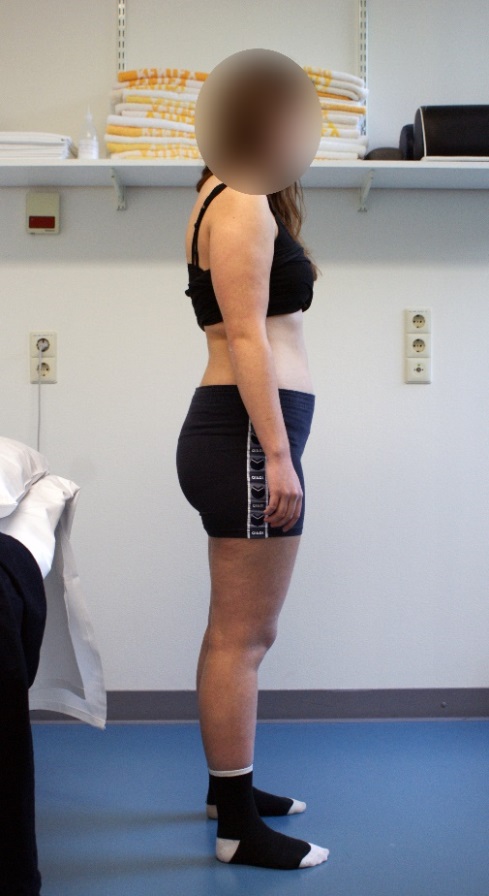


Incorrect

- Waiter’s bow: in standing position with knees slightly flexed, the patient is asked to bend forward from the hips, without flexing the lumbar spine. The test is graded as "correct" if the patient is able to bend forward at least 50^o^ of hip flexion without flexing the lumbar.
-
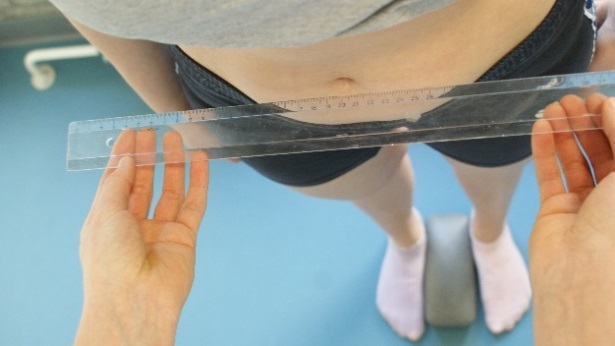

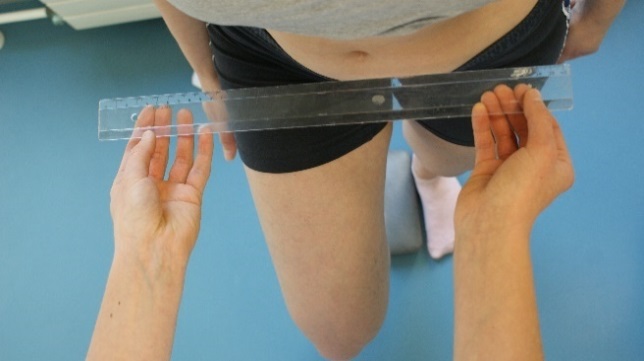
One leg stance: in standing position, with feet 10 cm apart, the patient is asked to lift one leg at a time. The test is graded as "correct" if the patient is able to lift both legs, the lateral movement of the belly button is less than 10cm and differs no more than 2 cm between sides.


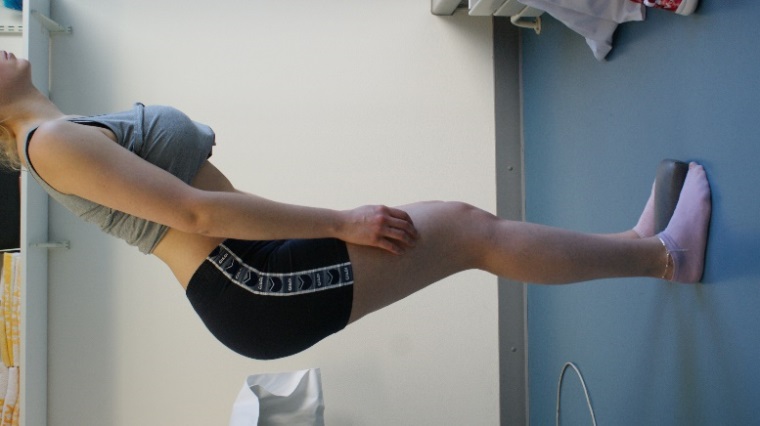


Correct


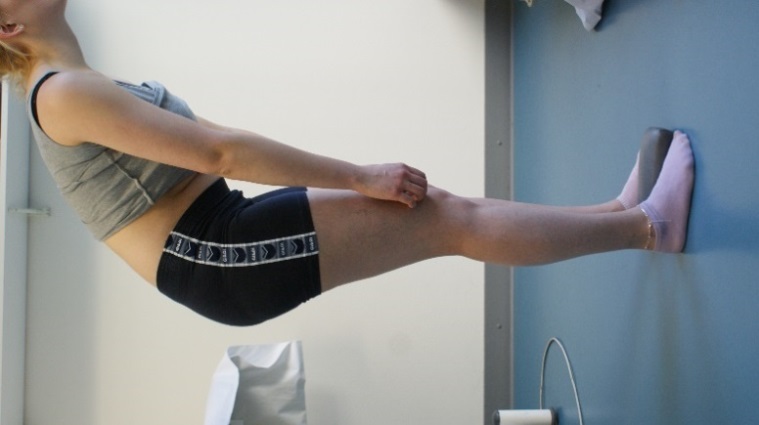


Incorrect

Ad II) *Muscle strength of the lumbar spine*:

-
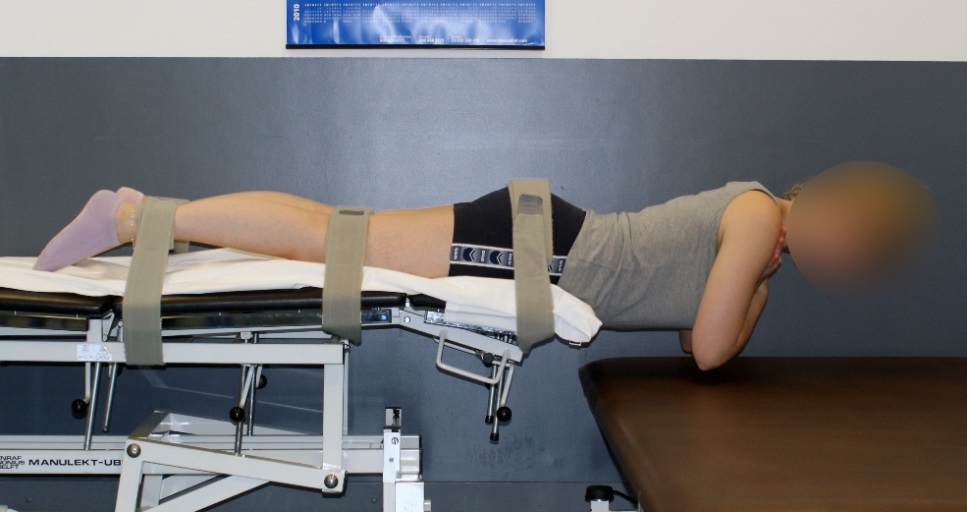
Sorensen test: This test is administered to test lumbar muscle endurance strength.^2^ During the test the patient lies on the examining table in a prone position with the upper edge of the iliac crests aligned with the edge of the table.^3^ The lower body is fixed to the table by three straps located around the pelvis, knees and ankles. With the arms folded across the chest, the patient is asked to isometrically maintain the upper body in a horizontal position.^3,4^ The patient must hold the test position for as long as possible, with a maximum of 240 seconds.^2^ This test is quick and easy to perform and might hold predictive value for LBP patients. This test also has good reliability (ICC= 0.77-0.88).^2,4^

Ad III) *Aerobic capacity*: To measure aerobic capacity the steep ramp test is used.^5,6^ The steep ramp test is a maximal exercise test on an exercise bicycle, able to predict VO_2_ max, which is the gold standard for measuring aerobic capacity. The steep ramp test predicts VO_2_ max more accurately than submaximal tests, and is highly correlated with VO_2_ max in different populations (Pearson r=0.82-0.85).^6^ Moreover, it is very reliable (ICC=0.996), easily administered, costs little time (8-12 minutes) and has little chance of complications in patients with low physical fitness.^5-7^ The outcome from the test will provide an estimation maximum short-time exercise capacity (Watt/kilograms). The test is performed on a LODE ergometer (Lode Corival, Lode BV, Groningen, the Netherlands). The protocol used, has a continues increase of 25 watt per 10 seconds.

Ad IV) *Flexibility:* To measure flexibility of the spine the finger floor distance is used. During the test the patient stands in a comfortable position with the feet 10 cm apart. The patient is asked to bend forward as far as possible while maintaining the knees, arms, and fingers fully extended.^8^ The vertical distance between the tip of the middle finger and the floor is measured with a ruler (in centimeters). This test has excellent validity, is highly correlated with trunk flexion assessed by radiologic measure (r = −0.96) and has excellent reliability (ICC = 0.99).^8^

Ad IV) *Mobility:* To measure mobility of the patient, the DEMMI and the Timed up and Go are used.

- DEMMI: The DEMMI measures mobility across the spectrum from bed bound to independent mobility.^9^ The DEMMI measures a person’s mobility on a scale from 0 to 19, with 19 indicating complete independent mobility. It is administered by clinician observation of performance on 15 hierarchical mobility challenges (from bed to walk and jump). Each item scored from 0 (unable) to 2 (independent). It has excellent validity and has fair reliability.^10,11^
- Timed up and Go: the Timed up and Go (TUG) measures time, in seconds, to stand up from a regular chair, walk a 3-m distance at a fast pace, turn around, return to the chair, and sit down again.^12^ The TUG is reliable (ICC= 0.80) and valid for the orthopedic population.^13^

^
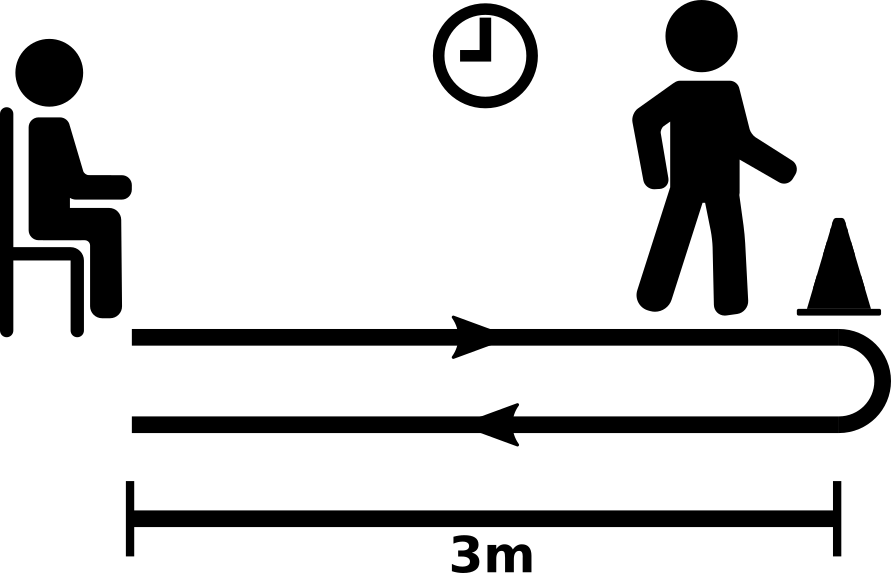
^

**References:**

1. Luomajoki H, Kool J, de Bruin ED, et al. Reliability of movement control tests in the lumbar spine. BMC Musculoskeletal Disord. 2007;8(1):1.
2. Demoulin C, Vanderthommen M, Duysens C, et al. Spinal muscle evaluation using the Sorensen test: a critical appraisal of the literature. Joint Bone Spine. 2006;73(1):43-50.
3. Latimer J, Maher CG, Refshauge K, et al. The reliability and validity of the Biering-Sorensen test in asymptomatic subjects and subjects reporting current or previous nonspecific low back pain. Spine. 1999;24(20):2085-9; discussion 90.
4. Luoto S, Heliövaara M, Hurri H, et al. Static back endurance and the risk of low-back pain. Clin Biomech (Bristol, Avon). 1995;10(6):323-4.
5. De Backer IC, Schep G, Hoogeveen A, Vreugdenhil G, Kester AD, van Breda E. Exercise testing and training in a cancer rehabilitation program: the advantage of the steep ramp test. Arch Phys Med Rehabil. 2007;88(5):610-6.
6. Rozenberg R, Bussmann J, Lesaffre E, et al. A steep ramp test is valid for estimating maximal power and oxygen uptake during a standard ramp test in type 2 diabetes. Scand J Med Sci Sports. 2015;25(5):595-602.
7. Meyer K, Samek L, Schwaibold M, et al. Interval training in patients with severe chronic heart failure: analysis and recommendations for exercise procedures. Med Sci Sports Exerc. 1997;29(3):306-12.
8. Perret C, Poiraudeau S, Fermanian J, et al. Validity, reliability, and responsiveness of the fingertip-to-floor test. Arch Phys Med Rehabil. 2001;82(11):1566-70.
9. de Morton NA, Davidson M, Keating JL. The de Morton Mobility Index (DEMMI): an essential health index for an ageing world. Health and Quality of Life Outcomes 2008, 6:63
10. Soares Menezes KVR, Auger C, de Souza Menezes WR, Guerra RO. Instruments to evaluate mobility capacity of older adults during hospitalization: A systematic review. Arch Gerontol Geriatr. 2017 Sep;72:67-79. doi: 10.1016/j.archger.2017.05.009. Epub 2017 May 27. PMID: 28599140.
11. Braun T., Schulz R.J., Reinke J., van Meeteren N.L., de Morton N.A., Davidson M., , Gruneberg C.: Reliability and validity of the German translation of the de Morton Mobility Index (DEMMI) performed by physiotherapists in patients admitted to a sub-acute inpatient geriatric rehabilitation hospital. BMC Geriatrics 2015; pp. 15.
12. Mathias S, Nayak US, Isaacs B. Balance in elderly patients: the “get-up and go” test. Archives of physical medicine and rehabilitation. 1986 Jun;67(6):387-389.
13. Yeung T.S., Wessel J., Stratford P.W., MacDermid J.C.: The timed up and go test for use on an inpatient orthopaedic rehabilitation ward. Journal of Orthopaedic & Sports Physical Therapy 2008; 38: pp. 410-417.
